# Impact of Compound Organic Fertilizer–Plant Combined Remediation on Microbial Community Structure in Mine Tailings Substrates

**DOI:** 10.3390/toxics14040285

**Published:** 2026-03-27

**Authors:** Tong Wu, Yan Bao, Yang-Chen Su, Teng-Da Yang, Xiao-Yun Leng, Chun-Fang Shi

**Affiliations:** College of Life Science and Technology, Inner Mongolia University of Science and Technology, Baotou 014010, China; lebront23@163.com (T.W.); 13113542251@163.com (Y.B.); suyc0817@163.com (Y.-C.S.); 15049301554@163.com (T.-D.Y.)

**Keywords:** compound organic fertilizer–plant combined remediation, soil microorganisms, ecological remediation, microbial community structure

## Abstract

Ecological restoration is increasingly applied as an effective strategy for mitigating environmental risks associated with tailings impoundments. However, plant establishment and ecological recovery in tailings substrates are often limited by unfavorable physicochemical properties and potential toxicity. This study investigated the changes in soil microbial community structure and diversity under the synergistic remediation of compound organic fertilizer and plants. Field plots subjected to combined organic fertilizer–plant remediation in a tailings impoundment in northern China were selected. The high-throughput sequencing of bacterial 16S rRNA genes and fungal ITS regions was performed alongside analyses of soil physicochemical properties. Compared to the untreated tailings soil, remediated soils showed pH values closer to neutrality, lower electrical conductivity, and significantly higher organic matter content, indicating an overall reduction in environmental stress and potential toxicity. The relative abundance of copiotrophic bacteria, such as Proteobacteria, increased, whereas that of stress-tolerant taxa adapted to extreme environments, such as Firmicutes, decreased. Although slight variations in dominant groups were observed among plots with different plant species, key microbial groups contributing to soil environmental improvement were largely consistent. These findings demonstrate that this combined remediation effectively improves the physicochemical properties and microbial community structure of tailings soil, providing a risk-oriented and ecologically sustainable strategy for the ecological restoration of similar sites.

## 1. Introduction

The massive waste generated during mineral processing, when stored long-term in tailings ponds, has become one of the most prominent environmental issues worldwide. More critically, most tailings ponds are often simply capped or filled in after mining operations conclude, lacking systematic ecological remediation. This leads to the continuous release of pollutants, causing long-term impacts on surrounding ecosystems [1]. Moreover, the fine particles of tailings are easily dispersed by wind, causing widespread atmospheric dust pollution [2]. Simultaneously, under the influence of precipitation and surface runoff, heavy metals enriched in tailings may undergo leaching and migration, entering soil and water systems before spreading to downstream environments, triggering transboundary ecological risks [3]. To alleviate the ecological and environmental pressures posed by tailings ponds, extensive research on remediation technologies has been conducted both domestically and internationally. Currently, the primary remediation methods primarily encompass physical, chemical, and biological approaches [4,5,6]. Physical remediation techniques such as thermal treatment, engineered cover, and electroremediation can rapidly improve substrate conditions and suppress pollutant dispersion by altering the physical structure of tailings or blocking migration pathways. Chemical remediation, on the other hand, triggers chemical reactions such as oxidation–reduction, precipitation, and adsorption by adding modifiers to regulate the form and mobility of heavy metals. This approach offers the advantages of rapid effectiveness and thorough treatment [7,8]. However, both approaches commonly suffer from high costs, significant disturbance, and potential disruption in in situ ecological structures. They may also cause secondary pollution and greenhouse gas emissions, limiting their large-scale implementation [9]. In contrast, ecological remediation, as a core approach to restoring damaged ecosystem functions, is gradually becoming the primary trend in tailings pond management. Phytoremediation is regarded as a sustainable remediation strategy that can replace traditional physicochemical methods due to its environmental friendliness, low operational costs, and high social acceptability [10,11]. As a vital component of bioremediation, phytoremediation primarily relies on plants and their rhizosphere microbial systems to absorb, immobilize, or transform pollutants such as heavy metals through mechanisms including phytoextraction, phytostabilization, and rhizofiltration [12]. This technology offers advantages such as low investment, simple maintenance, and minimal disruption to the landscape, making it particularly suitable for the long-term ecological restoration of large-scale, low-concentration contaminated tailings ponds [13]. However, the practical effectiveness of phytoremediation is significantly constrained by vegetation establishment rates and the growth performance of remediation plants. Successful implementation depends on suitable climatic conditions, the physicochemical properties of the substrate, agronomic management practices, and the selection and configuration of key restoration species [14]. Therefore, achieving the rapid establishment and stable growth of plant communities in the extreme habitats of tailings remains a critical scientific challenge that urgently requires breakthroughs in the field of tailings ecological restoration.

Studies have shown that the application of organic fertilizers can effectively improve the physical structure of tailings substrates by reducing bulk density, increasing porosity and water-holding capacity, and promoting the formation of aggregates, thereby enhancing structural stability. Additionally, organic fertilizers can regulate tailings pH to an appropriate near-neutral range through acid–base buffering, adsorption, complexation, and precipitation processes while effectively immobilizing the bioavailability of heavy metals [15]. Other studies have indicated that sludge and manure can raise the supply levels of essential elements in tailings to levels close to, or even higher than, those of natural soils, significantly improving plant nutrient use efficiency and thereby accelerating vegetation reconstruction and the restoration of ecological function [16]. However, despite the significant effects of organic fertilizers on improving the physicochemical properties of tailings and enhancing nutrient supply, the aforementioned remediation effects largely depend on the active involvement of microbial communities. Microorganisms are not only key drivers of soil structure and functional development but also core participants in organic matter transformation, nutrient cycling, and the regulation of heavy metal speciation. Currently, systematic research remains limited regarding how organic fertilizers systematically influence the structure and function of tailings microbial communities and their dynamic responses during vegetation restoration, particularly with respect to comparing differences and synergistic mechanisms among microbial communities under different remediation treatments, such as amended versus unamended substrates and various vegetation types.

Therefore, this study focuses on the response mechanisms of microbial communities to organic fertilizer amendment in tailings remediation. By comparatively analyzing the bacterial and fungal community characteristics of substrates amended with compound organic fertilizers and unamended substrates, as well as those of amended substrates planted with different plant species versus unamended substrates, this study aims to systematically elucidate the patterns of change in microbial community structure and function under organic fertilizer regulation, thereby providing a theoretical basis and practical support for a deeper understanding of microbially driven mechanisms in tailings ecological remediation.

## 2. Materials and Methods

### 2.1. Experimental Materials

#### 2.1.1. Soil Samples

Soil samples were collected from a demonstration site for compound organic fertilizer–plant combined remediation at a tailings pond in northern China. Six treatments were established: CK (no compound organic fertilizer), DT1 (compound organic fertilizer + sandwort), DT2 (compound organic fertilizer + ryegrass), DT3 (compound organic fertilizer + sedum), DT4 (compound organic fertilizer + ice plant), and DT5 (compound organic fertilizer + astragalus). After a one-time application of compound organic fertilizer and the transplanting of remediation plants, soil samples were collected from the 0–20 cm topsoil layer using the quartering method, then air-dried, ground, and sieved for further determination.

#### 2.1.2. Compound Organic Fertilizers

The compound organic fertilizer consists of organic fertilizer derived from surplus sludge and organic fertilizer derived from Chinese herbal medicine residues (2:1 by mass ratio). The organic fertilizer sourced from Chinese herbal medicine residues is provided by Jilin Wantong Group Shengtai Bioengineering Co., Ltd., Tonghua, China, while the organic fertilizer from surplus sludge is supplied by Shenyang Dongyuan Environmental Technology Co., Ltd., Shenyang, China.

### 2.2. Experimental Methods

#### 2.2.1. Determination of Physical and Chemical Properties of Tailings Substrate

The physicochemical properties of the tailings substrate, including soil moisture content, pH, electrical conductivity (EC), and organic matter (SOM), were determined using standard methods.

Soil moisture content was determined using the oven-drying method. Fresh soil samples were weighed, placed in aluminum boxes, and dried in a constant-temperature oven at 105 °C for 12 h until a constant weight was obtained. The moisture content was calculated based on the mass difference before and after drying.

Soil pH was measured in a 1:2.5 (soil:water, *w*/*v*) suspension. After thorough shaking and equilibration for 30 min, the pH value was determined using a calibrated multi-parameter analyzer (Model DZS-708T, LeiCi, INESA Scientific Instrument Co., Ltd., Shanghai, China).

Soil electrical conductivity (EC) was determined in the same 1:2.5 (soil:water, *w*/*v*) suspension as used for pH measurement, using the same multi-parameter analyzer (Model DZS-708T, LeiCi, INESA Scientific Instrument Co., Ltd., Shanghai, China) after equilibration.

Soil organic matter (SOM) was determined using the potassium dichromate oxidation–colorimetric method with external heating [17]. Soil samples were digested with a K_2_Cr_2_O_7_–H_2_SO_4_ solution at high temperature. Absorbance was measured using a spectrophotometer, and SOM content was calculated based on the consumption of Cr^6+^.

#### 2.2.2. Soil Microbial Community Structure

An amplifier sequencing analysis of soil microorganisms was performed by amplifying the V4 region of soil bacterial 16S rRNA using primers 515F and 806R. The workflow included DNA extraction, PCR amplification (V4 region), index addition, library quality control, sequencing, and final bioinformatic analysis to obtain the results. The amplification of the ITS1 region in soil fungi was performed using the ITS1-5F primer through the following steps: DNA extraction, PCR amplification, library construction, machine sequencing, and final bioinformatic analysis to obtain the results. Soil samples were sent to Shenzhen Wekemo Technology Group Co., Ltd., Shenzhen, China, where the amplicon sequencing data were analyzed via the Wekemo Bioincloud platform.

## 3. Results

### 3.1. Soil Physicochemical Properties

**Figure 1:**
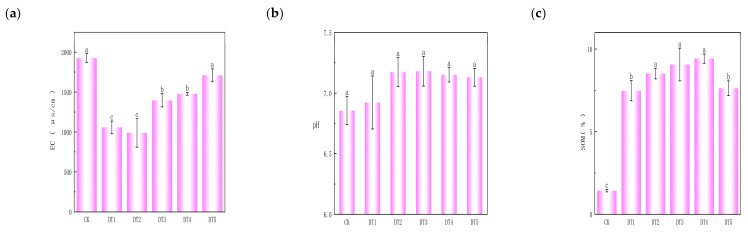
Soil physicochemical properties: (**a**) soil electrical conductivity map; (**b**) soil pH map; and (**c**) soil organic matter map. (Different lowercase letters (a, b, c, etc.) indicate significant differences among treatments at *p* < 0.05 level.)

The soil physicochemical properties of the six amended treatment plots in the study area are shown in Figure 1. There was no significant difference in pH between the improved soil and the tailings substrate. The electrical conductivity of the unamended tailings substrate was significantly higher than that of the amended and remediated soil. The organic matter content of the unamended tailings substrate was only 1.4%, which was significantly lower than that of the amended and remediated soil; following amendment and remediation, soil organic matter increased by 7.1–9.7%.

### 3.2. Community Structure of Microorganisms

#### 3.2.1. Microbial Community Structure Composition Diagram

**Figure 2:**
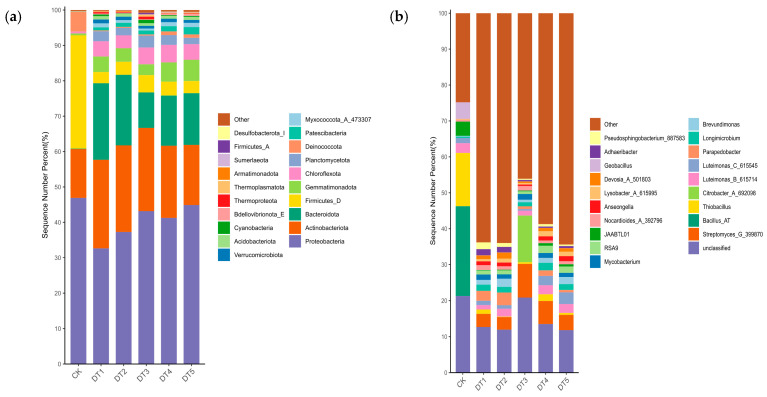
Soil bacterial community structure composition: (**a**) phylum-level bacterial community structure composition and (**b**) genus-level bacterial community structure composition.

**Figure 3:**
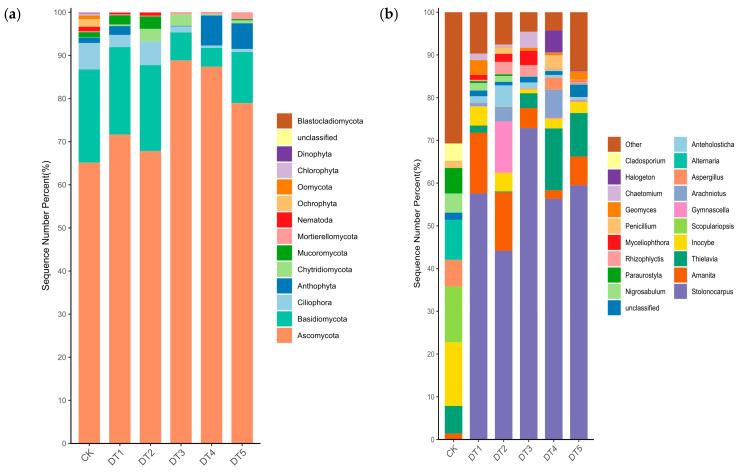
Soil fungal community structure composition: (**a**) fungal community structure composition at the phylum level and (**b**) fungal community structure composition at the genus level.

For each sample, the top 20 taxa in terms of relative abundance at the phylum and genus levels were selected to generate relative abundance bar charts (Figure 2 and Figure 3). At the phylum level of the bacterial community, Proteobacteria dominated across all groups, followed by Actinobacteriota. Bacteroidota was detected only in the experimental groups. The relative abundance of Firmicutes was significantly reduced in the amended soil. At the genus level of the bacterial community, *Bacillus* AT and *Thiobacillus* were enriched exclusively in the CK group, whereas *Streptomyces* G_399870 was enriched only in the amended soil.

At the phylum level of the fungal community, Ascomycota dominated across all groups, with higher relative abundance in the experimental groups than in the CK group, reaching a peak in DT3. Basidiomycota was enriched in all groups but exhibited the highest relative abundance in the CK group. At the genus level of the fungal community, *Stolonocarpus* dominated in the experimental groups but was not enriched in the CK group. *Amanita* was enriched across all groups, with increased relative abundance in the experimental groups, whereas *Inocybe* was also enriched in all groups but exhibited higher relative abundance in the CK group.

#### 3.2.2. Microbial Venn Diagram

**Figure 4:**
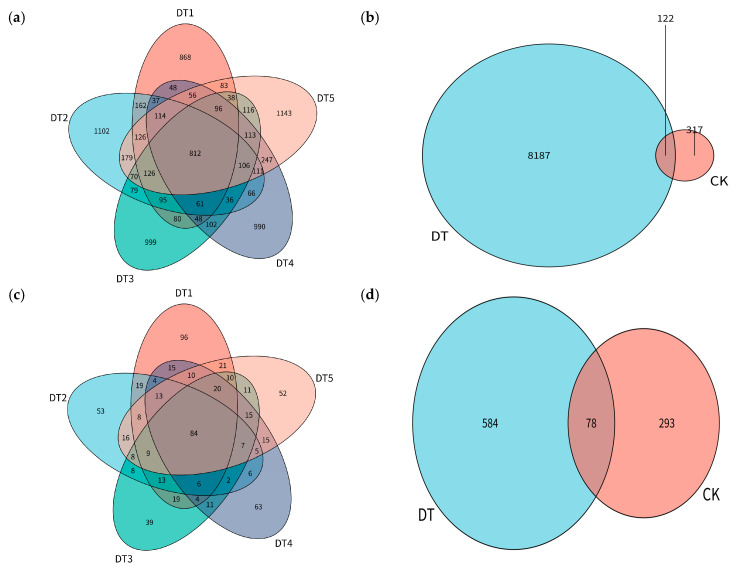
Soil bacterial and fungal petal diagrams and Venn diagrams: (**a**) bacterial petal diagram; (**b**) bacterial Venn diagram; (**c**) fungal petal diagram; and (**d**) fungal Venn diagram.

Venn diagram analysis further revealed the overlap of bacterial and fungal OTUs among different samples. OTU analysis indicated (Figure 4) that the bacterial OTU counts for the DT1, DT2, DT3, DT4, and DT5 treatments were 2850, 3282, 2977, 3043, and 3178, respectively. The five experimental groups shared 812 bacterial OTUs, accounting for 28.49%, 24.74%, 27.27%, 26.68%, and 25.55% of their respective OTUs; the group-specific bacterial OTUs were 868, 1102, 999, 990, and 1143, respectively, accounting for 30.46%, 33.58%, 32.83%, 32.53%, and 35.97% of their respective OTUs. DT5 exhibited 5.51% more group-specific bacterial OTUs than DT1. The DT and WK groups shared 95 bacterial OTUs, while CK contained 344 unique OTUs, and DT contained 4335 unique OTUs.

The fungal OTU counts for the DT1, DT2, DT3, DT4, and DT5 treatments were 353, 261, 266, 270, and 304, respectively. The five experimental groups shared 84 fungal OTUs, accounting for 23.8%, 32.18%, 31.58%, 31.11%, and 27.63% of their respective fungal OTUs. The number of fungal OTUs unique to each group was 96, 53, 39, 63, and 52, respectively, accounting for 27.2%, 20.31%, 14.66%, 23.33%, and 17.11% of their respective OTUs. This indicates that the DT5 treatment increased both the total number of bacterial and fungal OTUs in the remediated tailings soil and the number of unique bacterial and fungal OTUs specific to each treatment. DT and CK shared 51 bacterial OTUs, with CK harboring 320 unique bacterial OTUs and DT having 336 unique bacterial OTUs.

#### 3.2.3. Microbial Clustering Heatmap Results

**Figure 5:**
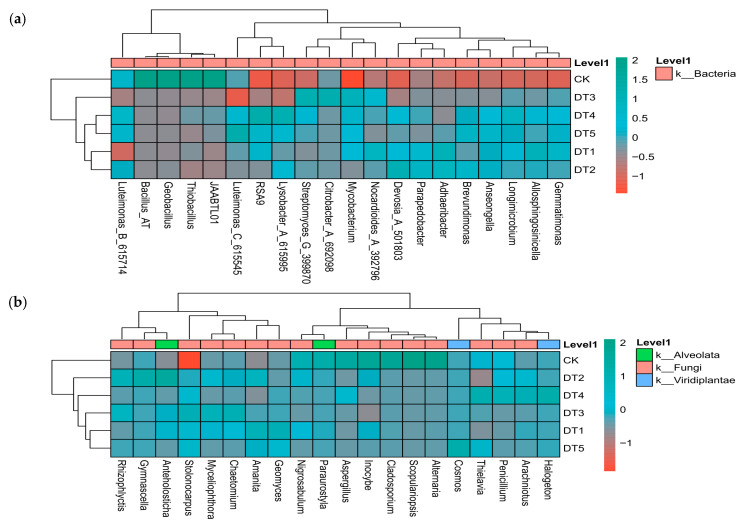
Heatmaps of soil bacterial and fungal clustering: (**a**) heatmap of bacterial genus-level clustering and (**b**) heatmap of fungal genus-level clustering.

A clustering heatmap analysis of bacterial and fungal communities (Figure 5) revealed samples clustering into two major groups: the control group (CK) and the experimental groups (DT1–DT5). Bacterial communities can be divided into two major categories: one dominated by *JAABTL01*, *Thiobacillus*, *Geobacillus*, *Bacillus* AT, and *Luteimonas* B_615714, which exhibit higher relative abundances in CK plots, and another characterized by *Streptomyces* G_399870, *Lysobacter* A_615995, and *Luteimonas* C_615545, which exhibit higher relative abundances in the amended plots. Within the fungal community, fungi such as *Alternaria*, *Scopulariopsis*, and *Inocybe* show higher relative abundances in the CK plots, while *Amanita*, *Stolonocarpus*, and *Anteholosticha* were more abundant in the amended plots.

### 3.3. Results of Microbial Diversity

#### 3.3.1. Microbial Beta Diversity

**Figure 6:**
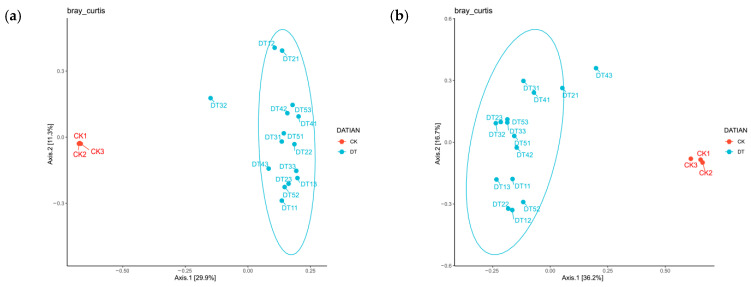
PCoA plots of soil bacteria and fungi: (**a**) principal component analysis of bacteria; (**b**) principal component analysis of fungi.

The score plot of the PCoA based on Bray–Curtis distance (Figure 6) reveals that the distribution patterns of the amended plot samples differ significantly from those of the CK treatment. For bacterial distribution patterns (Figure 6a), CK samples cluster in the negative quadrant, while DT samples cluster in the positive quadrant. For fungal distribution patterns (Figure 6b), CK samples cluster in the right quadrant, whereas DT samples cluster in the upper-left region.

#### 3.3.2. Microbial Alpha Diversity

**Figure 7:**
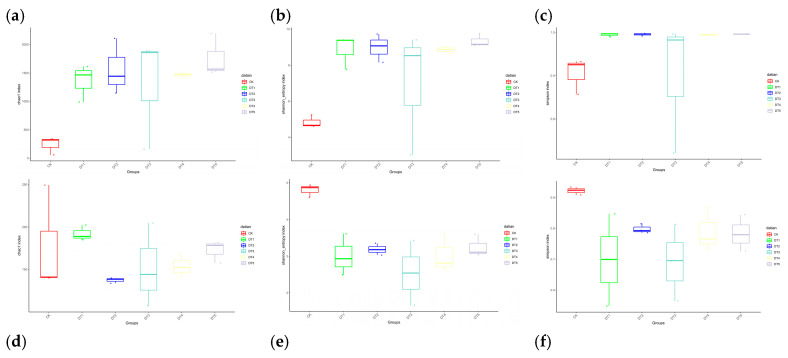
Alpha diversity of soil bacteria and fungi: (**a**) bacterial Chao1 diversity index; (**b**) bacterial Shannon diversity index; (**c**) bacterial Simpson diversity index; (**d**) fungal Chao1 diversity index; (**e**) fungal Shannon diversity index; and (**f**) fungal Simpson diversity index.

This study evaluated the α-diversity characteristics of soil bacterial and fungal communities (Figure 7). The results indicated that the Chao, Shannon, and Simpson indices of soil bacterial communities in the amended plots were higher than those in the CK group. Regarding soil fungal community diversity, the Shannon and Simpson indices of CK were higher than those of other treatment groups, while the Chao1 indices of DT1 and DT5 were higher than those of other treatment groups and CK.

### 3.4. Correlation Analysis Between Dominant Species in Soil Microbial Communities and Soil Physicochemical Properties and Enzyme Activity

**Figure 8:**
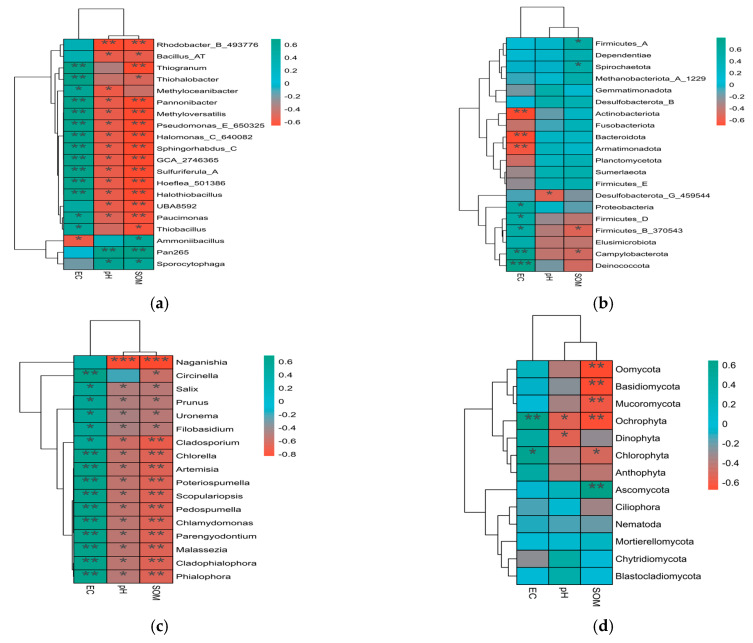
Correlation analysis between relative abundance of dominant bacterial and fungal groups and physicochemical properties: (**a**) correlation analysis at bacterial genus level; (**b**) correlation analysis at bacterial phylum level; (**c**) correlation analysis at fungal genus level; and (**d**) correlation analysis at fungal phylum level. Notes: *, **, and *** indicate significant correlations at *p* < 0.05, *p* < 0.01, and *p* < 0.001 levels, respectively.

Among dominant bacterial microbial species (Figure 8), Firmicutes showed a significant positive correlation with electrical conductivity, while Actinobacteriota exhibited a highly significant negative correlation. *Sporocytophaga* demonstrated a significant positive correlation with pH, while Bacillus AT exhibited a significant negative correlation. *Bacillus* AT and *Thiobacillus* showed a highly significant negative correlation with organic matter content, whereas *Sporocytophaga* displayed a significant positive correlation.

Among dominant fungal microbial species (Figure 8), *Ochrophyta* showed a highly significant positive correlation with electrical conductivity. *Ochrophyta* exhibited a significant negative correlation with pH, while Naganishia showed an extremely significant negative correlation. *Ascomycota* demonstrated a highly significant positive correlation with organic matter, whereas Poteriospumella and Scopulariopsis exhibited highly significant negative correlations.

## 4. Discussion

### 4.1. Improvement in the Soil Environment by Combined Remediation with Compound Organic Fertilizer and Plants

The application of compound organic fertilizer provides abundant nutrients and a favorable growth environment for microorganisms, driving soil pH toward neutrality and enhancing soil fertility. Against the background that the pH of tailings was generally adjusted to a neutral range following organic fertilizer amendment, distinct plant species formed differentiated pH microenvironments in the rhizospheric microdomain via root physiological metabolism and interactive effects with rhizosphere microorganisms. Specifically, the root activities of Agriophyllum squarrosum tended to maintain or slightly acidify the rhizosphere, thereby resulting in a measured pH value that was closer to the unamended background (6.8). In contrast, the root activities of plants such as Lolium perenne exhibited a tendency to alkalinize the rhizosphere, leading to a slight elevation in pH (7.2). Although such variations were within the overall neutral pH range, they explicitly reflected the species-specific characteristics of plant–soil interactions. These species-specific differences in rhizosphere pH are presumably associated with variations in root exudate compositions (e.g., organic acids, protons, or hydroxyl ions) among different plant species, as well as the functional diversity of rhizosphere microbial communities modulated by root exudation and root-associated physiological activities [18]. Collectively, these findings further confirm that plant species selection exerts a pivotal role in shaping the rhizospheric microenvironment of tailings, even under the condition of overall neutral soil pH after organic fertilizer improvement, which provides an important theoretical basis for optimizing plant species selection in tailings vegetation restoration practices. Plant root systems significantly increased the abundance of microbial communities in the rhizosphere, thereby improving soil biological activity. These findings are consistent with the results reported by Liao K et al. [19] and Zhang H et al. [20]. Soil physicochemical properties directly influence the structure and diversity of soil microbial communities. As soil remediation progresses, increases in pH and organic matter content in tailings directly lead to a significant enhancement in microbial α-diversity [21]. Concurrently, the decline in salinity and the associated reduction in electrical conductivity facilitate the gradual succession of microbial communities toward greater complexity [22]. The results of this study further demonstrate that, following combined remediation with compound organic fertilizer and plants, soil organic matter content significantly increased, while the relative abundances of *Bacillus* AT and *Thiobacillus*, which were significantly negatively correlated with organic matter, declined markedly. Previous studies have shown that in acidic tailings during natural succession or the early stage of restoration, the occurrence of *Bacillus* AT and *Thiobacillus* is often accompanied by the rapid depletion of organic matter. These taxa typically exhibit higher relative abundances in oligotrophic environments and are considered oligotrophic microorganisms [23,24]. Therefore, combined remediation using compound organic fertilizer and plants not only enhances soil organic matter content and improves soil pH and electrical conductivity but also promotes the growth of beneficial microorganisms and optimizes the structure of soil microbial communities.

### 4.2. Effects of Combined Remediation with Compound Organic Fertilizer and Plants on Microbial Community Structure of Tailings Substrates

#### 4.2.1. Dominant Microbial Taxa in Amended Soils

In the soil bacterial community, Proteobacteria, Bacteroidetes, and Actinobacteria were identified as the dominant phyla in the amended soil (Figure 2), indicating that the nutrient-enriched soil condition provided favorable habitats for these taxa. Proteobacteria are typically classified as copiotrophic bacteria [25] and are capable of promoting metal bioreduction in contaminated systems through the regulation of microbial diversity, thereby exhibiting considerable remediation potential in polluted soils [26]. Bacteroidetes can colonize various agriculturally important crops [27,28,29,30]; for example, the relative abundance of Bacteroidetes in the rhizosphere of *Arabidopsis thaliana* increases with plant growth [31]. Actinobacteria contribute to enhanced plant tolerance under high-salinity conditions and promote plant growth [32]. At the bacterial genus level, *Streptomyces* was enriched only in the amended soils. This enrichment can be attributed to the ability of *Streptomyces* to modify rhizosphere microbial communities, particularly by promoting microorganisms beneficial to plant growth [9]. Similar to Fang’s [33] research, *Streptomyces* exhibits favorable growth under low contaminant concentrations in soil, whereas its growth performance declines as contaminant concentrations increase.

Compared to bacterial communities, fungal community structure remained relatively stable throughout the remediation process (Figure 3). Ascomycota dominates all treatment groups, reflecting their role as the primary decomposers in soil ecosystems [34]. Members of Ascomycota possess a greater abundance of genes associated with stress tolerance and resource acquisition, indicating a superior capacity to colonize diverse environments compared with other fungal phyla [12,13]. Moreover, Ascomycota consistently represents the most prevalent phylum at metal-contaminated sites, accounting for 97–99% of all root-associated fungi across grass species [35]. At the fungal genus level, *Stolonocarpus* was dominant in the experimental groups. *Stolonocarpus* is a newly established genus within Ascomycota, whose taxonomic status emerged from a phylogenetic reassessment of the polyphyletic genus *Thielavia*. In amended soils, the relative abundance of *Stolonocarpus* reached around 50%, whereas its abundance in the unamended soil was nearly zero. This shift can be attributed to the inherently poor structure and unfavorable physicochemical properties of tailings soils, such as pH imbalance and nutrient deficiency. During remediation, the application of soil amendments and the establishment of vegetation improved soil pH, enhanced soil fertility, and increased water- and nutrient-holding capacity, thereby creating a more suitable growth environment for *Stolonocarpus* and facilitating its proliferation. The genus *Thielavia* was significantly enriched across all treatment groups, consistent with the composting study reported by Montiel-Rozas et al. [36]. This genus exhibits a strong positive correlation with soil organic matter content and plays a central functional role in organic carbon decomposition and humification processes driven by organic fertilizer inputs.

#### 4.2.2. Dominant Microbial Taxa in Tailings Substrates

Following combined remediation, the tailings substrate was substantially improved, accompanied by a marked decline in the relative abundance of certain microorganisms adapted to extreme environments. In the bacterial community, Firmicutes accounted for nearly 30% of the total relative abundance in the CK group but decreased to approximately 5% in the amended soil. Due to their abilities to cope with extreme conditions by upregulating genes associated with dormancy and sporulation [37], and their resistance to high concentrations of heavy metals, the species maintains relatively high abundance in contaminated soils, indicating the presence of physiological mechanisms conferring tolerance to metal toxicity and establishing Firmicutes as a dominant taxon in extreme soil environments [38]. Arunrat et al. [39,40] similarly reported an increase in Firmicutes abundance following summer fires in agricultural soils, followed by declines during the rainy season and winter, consistent with the findings of this study. Several stress-tolerant bacterial taxa, including *JAABTL01*, *Thiobacillus*, *Geobacillus*, and *Bacillus* AT, occupied dominant ecological niches in the CK group, while their relative abundances decreased sharply by 50–90% across the remediation treatments. Among these, *JAABTL01* is a heavy metal-tolerant microorganism capable of maintaining membrane transport and oxidative stress homeostasis under combined Cd and As stress, thereby retaining a competitive advantage [41]. *Thiobacillus* is an obligate acidophilic and chemolithoautotrophic genus that derives energy from the redox transformations of inorganic sulfur and occupies a critical geochemical niche in low-pH, carbon-limited tailings environments [42]. *Geobacillus* maintains metabolic activity under high temperature or carbon limitation through the synthesis of heat shock proteins and compatible solutes and is regarded as a phylogenetically conserved stress indicator taxon [43]. *Bacillus* AT, in turn, relies on sporulation and osmotic regulation mechanisms to withstand osmotic and oxidative stress, thereby maintaining population stability in extreme habitats [44]. The combined application of organic fertilizer and plants significantly reduced heavy metal toxicity while increasing soil pH and organic matter content, rapidly constraining the ecological niches of these stress-tolerant taxa and providing strong evidence for the observed restructuring of the microbial community structure following amendment.

Within the fungal community, the relative abundance of *Scopulariopsis* in tailings substrates declined to nearly zero following soil remediation. Likewise, the high-abundance indicator taxa *Alternaria*, *Cladosporium*, and *Inocybe* exhibited declines of one to two orders of magnitude (a 90–99% decrease in relative abundance) after combined organic fertilizer–plant remediation. Previous studies indicate that during long-term adaptation to heavy metal environments, *Scopulariopsis* can develop specific metabolic pathways or resistance mechanisms. However, once metal toxicity is alleviated, this genus may lose its competitive advantage in interactions with other microorganisms, leading to a decline in relative abundance [45]. This interpretation is supported by the work of Gostinčar C et al. [46], who also found that *Scopulariopsis* exhibits strong adaptability to extreme conditions. *Alternaria* has been widely recognized as a heavy metal-tolerant genus [47,48], *Cladosporium* as a halotolerant and alkali-tolerant taxon [49], and Inocybe as an environmentally sensitive indicator fungus [50], all of which rely on extreme stress conditions to maintain ecological competitiveness. The remediation measures implemented in this study significantly reduced metal bioavailability, alleviated salinity and acidity stress, and enhanced the availability of organic matter and nutrients, thereby compressing the ecological space available to these stress-adapted fungi. Concurrently, rapidly growing copiotrophic taxa (such as Ascomycota and Bacteroidetes) further restricted their niches through resource competition and spatial exclusion. Therefore, the decline in these taxa can be regarded as an early mycological signal of ecological succession from an extreme tailings environment toward a nutrient-enriched soil ecosystem.

#### 4.2.3. Composition of Dominant Taxa Under Different Remediation Strategies

Slight differences in dominant microbial taxa were observed among plots planted with different vegetation species. In this study, the relative abundance of *Citrobacter* was higher in Carex-amended soil (DT3) than in other treatments. This is because of the greater abundance of amino acids and polypeptides in Carex root exudates. *Citrobacter* is well adapted to utilizing these complex nitrogen sources, whereas the rhizospheres of xerophytic plants (e.g., Agropyron and Salsola) lack these substrates, resulting in lower abundances of this genus [51]. In contrast, *Luteimonas* exhibited lower abundance in Carex-amended soils, which may be attributed to its ecological function and environmental preferences. *Luteimonas* is efficient at degrading complex plant polymers. Residual material from gramineous plants such as Agropyron and ryegrass is rich in recalcitrant compounds, providing suitable niches for this genus. In the Carex rhizosphere, however, organic matter is typically more labile or decomposed by other microbial groups [52]. Differences in dominant taxa were also observed between Salsola-amended soil (DT1) and Agropyron-amended soil (DT4). Previous studies have indicated that the Salsola rhizosphere is dominated by oligotrophic and stress-resistant taxa, such as Arthrobacter and Proteobacteria [50], whereas Agropyron tends to enrich microorganisms capable of degrading recalcitrant organic matter (e.g., Actinobacteria) to sustain long-term nutrient cycling, reflecting a strategy markedly different from that of annual plants like Salsola [53]. Similarly, differences between ryegrass-amended soil (DT2) and Cosmos-amended soil (DT5) were attributed to contrasting root exudate profiles: Cosmos releases higher levels of flavonoids, leading to an enrichment in *Lysobacter* and *Gemmatimonas*, whereas ryegrass exudes greater amounts of organic acids, favoring *Thiobacillus* and Acidobacteria [54].

In terms of fungal communities, the CK group was clearly separated from all treatment groups, indicating marked differences in species composition. The relative abundance of *Gymnascella* was higher in the ryegrass-amended soil (DT2) than in other treatment groups, likely because *Gymnascella* is a nitrogen-preferring fungus. When ryegrass cultivation is accompanied by the substantial application of organic fertilizers, fungi capable of utilizing readily decomposable organic nitrogen are preferentially enriched in the rhizosphere [55]. The relative abundance of Anthophyta was higher in Agropyron-amended soil (DT4) and Cosmos-amended soil (DT5) than in other treatments. As a perennial grass, Agropyron accumulates substantial root residues and litter, which increase soil organic matter and directly elevate the relative proportion of plant-derived DNA (Anthophyta) in soil [52]. Annual herbs like Cosmos contribute large amounts of pollen, seeds, and leaf residues at the end of the growing season, all of which are rich in plant DNA. In soil metagenomic analyses, these residues can significantly increase the sequencing reads for Anthophyta [56]. Differences in dominant fungal taxa were also observed between Salsola-amended soil (DT1) and Carex-amended soil (DT3): Salsola tended to enrich fungi characterized by short life cycles and high metabolic activity (e.g., Basidiomycota), whereas Carex favored fungi with longer life cycles and stable symbiotic associations (e.g., Ascomycota) [51,57].

### 4.3. Microbial Diversity

Bacterial α-diversity indices (Chao1, Shannon, and Simpson) were significantly lower in the CK group than in all remediation treatments (*p* < 0.05), indicating that species richness, evenness, and dominance distribution were all suppressed under the extreme tailings environment. This pattern is consistent with observations from other extreme ecosystems, including glacier forefields [58], hypersaline lakes [59], and the saline formation waters of oil reservoirs [60]. Amen et al. [58] found that early successional stages in glacier forefields are dominated by a single pioneer genus, with microbial interaction networks largely absent and community complexity approaching zero. Gao et al. [59] demonstrated that increasing salinity reduces species richness and simplifies co-occurrence networks through osmotic stress in saline lakes on the northern slope of the Tianshan Mountains. Similarly, Scheffer et al. [60] detected extremely low 16S rRNA diversity in formation waters from oil reservoirs in the Gulf of Mexico with salinity levels of 4.5%. Collectively, these studies indicate that high salinity, oligotrophic conditions, and heavy metal toxicity can synergistically suppress microbial diversity. In the present study, combined remediation using compound organic fertilizer and plants effectively improved soil conditions and enhanced resource heterogeneity, thereby driving a significant recovery of microbial α-diversity.

## 5. Conclusions

Compared with single physical or chemical remediation methods, combined remediation using compound organic fertilizer and plants demonstrates more pronounced integrated advantages in tailings ecological restoration. A one-time application of compound organic fertilizer can significantly increase soil organic matter and readily available nutrient contents, comprehensively improving the porosity, water-holding capacity, and pH buffering performance of tailings substrates, thereby providing a sustained and balanced nutrient reservoir for plant establishment. The subsequent establishment of plants with different ecological traits further reduces surface temperature fluctuations, suppresses the re-suspension of heavy metals, and drives microbial community succession toward a “copiotrophic–multifunctional” state through processes such as root inputs, rhizosphere deposition, and canopy shading, forming a positive feedback loop of synergistic enhancement among soil fertility, plants, and microorganisms. By increasing soil organic matter content and improving physicochemical properties, compound organic fertilizer–plant remediation successfully induces the positive succession of microbial communities. The decline in stress-tolerant taxa negatively correlated with organic matter, such as Thiobacillus and Bacillus AT, together with the enrichment in copiotrophic and functionally important taxa including Proteobacteria and Streptomyces, provides key microbiological evidence for the transformation of tailings substrates from an “extreme habitat” to “ecologically functional soil”. Therefore, compound organic fertilizer–plant combined remediation offers new theoretical support for the ecological restoration of tailings impoundments.

In the future, we will further investigate the improvement effect of compound organic fertilizer on mine tailings substrates, with a focus on the research and development of mass production technology for the “compound organic fertilizer–plant combined remediation package”. Our ultimate goal is to achieve the environmentally friendly and cost-effective remediation of large-area mine tailings substrates while promoting the resource utilization of wastes such as traditional Chinese medicine residues and municipal excess sludge and expanding the practical application scenarios of ecological remediation technologies.

## Data Availability

The original contributions presented in this study are included in the article. Further inquiries can be directed to the corresponding authors.
